# A New Retreat for the Insane

**Published:** 1858-05

**Authors:** 


*A New Retreat for the Insane.*—We would call the attention of our readers to the establishment of a new retreat for the insane, at Canandaigua, under the direction of Dr. Geo. Cook, a gentleman who is highly accomplished in this important and difficult branch of medical science, and has had a large experience in the New York State Lunatic Asylum. There could be no location for such an establishment more favorable than the village of Canandaigua, and physicians who commit their patients to Dr. Cook's care, will enjoy the advantages of the purest air, judicious treatment, and frequently a contiguity to the patient's residence, which is highly desirable.

